# Free-Standing Organic Transistors and Circuits with Sub-Micron Thicknesses

**DOI:** 10.1038/srep27450

**Published:** 2016-06-09

**Authors:** Kenjiro Fukuda, Tomohito Sekine, Rei Shiwaku, Takuya Morimoto, Daisuke Kumaki, Shizuo Tokito

**Affiliations:** 1 Research Center for Organic Electronics (ROEL), Graduate School of Science and Engineering, Yamagata University, 4-3-16 Jonan, Yonezawa, Yamagata, 992-8510, Japan; 2 Japan Science and Technology Agency, PRESTO, 4-1-8, Honcho, Kawaguchi, Saitama, 332-0012, Japan; 3 Thin-Film Device Laboratory, RIKEN, 2-1, Hirosawa, Wako, Saitama, 351-0198, Japan; 4 Center for Emergent Matter Science, RIKEN, 2-1 Hirosawa, Wako-shi, Saitama 351-0198, Japan; 5 Department of Mechanical, Electrical and Electronic Engineering, Shimane University, 1060 Nishikawatsu, Matsue, Shimane, 690-8504, Japan

## Abstract

The realization of wearable electronic devices with extremely thin and flexible form factors has been a major technological challenge. While substrates typically limit the thickness of thin-film electronic devices, they are usually necessary for their fabrication and functionality. Here we report on ultra-thin organic transistors and integrated circuits using device components whose substrates that have been removed. The fabricated organic circuits with total device thicknesses down to 350 nm have electrical performance levels close to those fabricated on conventional flexible substrates. Moreover, they exhibit excellent mechanical robustness, whereby their static and dynamic electrical characteristics do not change even under 50% compressive strain. Tests using systematically applied compressive strains reveal that these free-standing organic transistors possess anisotropic mechanical stability, and a strain model for a multilayer stack can be used to describe the strain in this sort of ultra-thin device. These results show the feasibility of ultimate-thin organic electronic devices using free-standing constructions.

Reducing the thickness of thin-film electronic devices has been a primary goal in the field of flexible electronics because it would give these devices the attributes of flexibility and lightness^1^. Electronic devices that are only a few microns thick can conform to the human body or other irregular surfaces^2^,^3^. Recent research has enabled the fabrication ultra-thin electronic devices with overall thicknesses of less than 10 μm and weights of less than 5 gm^−2^, and these devices have been demonstrated in a variety of applications such as integrated circuits^2^,^4^,^5^,^6^, light-emitting diodes^7^, solar cells^8^,^9^, and sensors^3^,^10^.

Recent studies have decreased the total thickness of flexible electronics to less than 1 μm^2^,^4^,^8^,^9^,^10^,^11^. Since even thinner films would have not only flexibility but also adhesiveness^12^, a continued reduction in the total thickness of flexible electronics is an important challenge toward the realization of wearable electronics. Although substrates are usually necessary during fabrication and for functionality, they limit the thickness of thin-film electronic devices. In attempts to reduce the total thickness, some researchers have devised free-standing electronic devices; the thin and free-standing dielectric layers in these devices are used as substrates and gate insulators at the same time^13^,^14^,^15^,^16^,^17^,^18^,^19^,^20^,^21^,^22^,^23^,^24^,^25^,^26^,^27^,^28^. While this would enable fabrication of extremely thin electronic devices, it is unknown to what extent substrate-free construction can reduce the overall device thickness or how stable these devices are under mechanical deformation.

In this study, we fabricated organic TFT devices and integrated circuits with total thicknesses as small as 350 nm by using free-standing construction and showed that these devices have a high degree of mechanical robustness. We also carried out precise evaluations of how the directionality of the mechanical strain affects the electrical performance of the devices and found an anisotropic dependency on the strain direction. Moreover, we analyzed the local strain applied to free-standing devices using a strain model for a four-layer device.

## Results

### Fabrication of free-standing Organic Transistors

**Figure 1:**
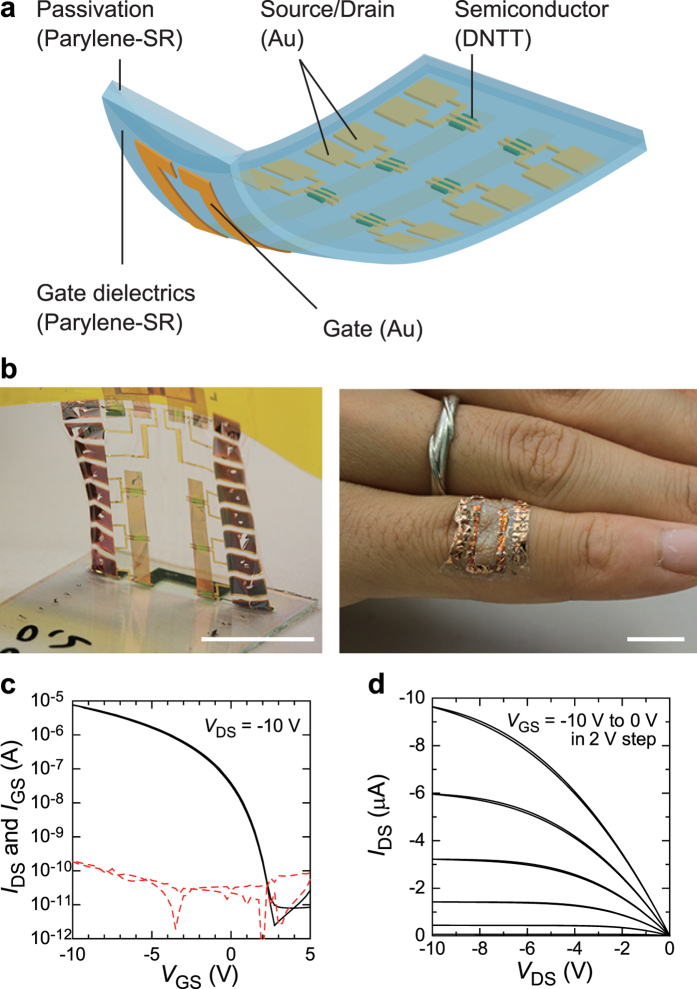
Free-standing organic thin-film transistors. (**a**) Schematic illustration of free-standing organic TFT devices. (**b**) Photograph showing the procedure for peeling the devices from the supporting glass carrier (left) and a device conforming to a human finger (right). Scale bars, 1 cm. (**c**) Transfer characteristics of organic TFT device with 100-nm-thick gate dielectric layer (total device thickness of 350 nm). The plot is of the drain-source current (*I*_DS_, solid black line) and gate leakage current (*I*_GS_, dashed red line) as a function of gate-source voltage (*V*_GS_) at a drain-source voltage (*V*_DS_) of −10 V. The saturation mobility is 0.37 cm^2^ V^−1^ s^−1^, and the on/off ratio exceeds six orders of magnitude. (**d**) Corresponding output characteristics. The plot is of *I*_DS_ as a function of *V*_DS_ for *V*_GS_ from 0 V to −10 V in 2 V steps.

We fabricated organic TFT devices with a bottom-gate and top-contact constructions on a 0.7-mm-thick glass plate with a Teflon™ release layer. After fabrication, the free-standing devices were peeled off from the supporting plate (Fig. 1a). Here, Parylene-SR was used to make the gate dielectric layer and passivation layer. Gold was used as the gate and source-drain layers, and dinaphtho[2,3-b:2′,3′-f]thieno[3,2-b]thiophene (DNTT) was used as the organic semiconducting layer^29^. Because of the low surface energy of Teflon™, the gold contacts and parylene-SR layers could be readily peeled from the supporting glass plate without being damaged. We prepared several devices with different dielectric layer thicknesses ranging from 100 nm to 250 nm. In order to improve their mechanical durability, some of the devices were uniformly encapsulated with parylene-SR passivation layers whose thicknesses were the same as those of the gate dielectric layers^1^,^30^. The details of the fabrication can be found in the Methods section and Supplementary Figure S1. The thinnest fabricated devices with passivation layers were only 350 nm thick and the dielectric and passivation layers were each 100 nm thick. The thickest fabricated devices were 650 nm, of which the dielectric and passivation layers were each 250 nm. When the devices were peeled from their supporting plates, the gate electrodes that had been directly evaporated onto the release layer were completely transferred to the gate dielectric layer (Fig. 1b) because the adhesive forces between the gold and Teflon™ release layer were sufficiently smaller than those between the gold and gate dielectric layers. Note that the electrical characteristics, including the gate leakage current, were at nearly the same levels before and after the devices were peeled from the supporting plates (Fig. S2). The resulting free-standing organic TFT devices were ultra-lightweight and ultra-thin, which would enable their unobtrusive conformation to the human body (Fig. 1b).

### Electrical Performance of Ultra-Thin Organic TFT Devices

The thinnest organic TFT devices (total thicknesses of 350 nm) functioned properly after being removed from the supporting plates (Fig. 1c). The estimated mobility in the saturation region (0.37 cm^2^ V^−1^ s^−1^), threshold voltage (−0.55 V), and on/off current ratio (exceeded 10^6^) obtained from transfer characteristics were comparable to those of organic TFT devices fabricated on rigid glass or flexible substrates^2^,^29^,^31^. The maximum gate leakage current was 200 pA, which indicates that the ultra-thin, free-standing gate dielectric layers provided excellent electrical insulation properties. The output characteristics (Fig. 1d) showed a proportional increase in the source-drain current (*I*_DS_) within the linear region, which indicates relatively low contact resistance between source/drain electrodes and semiconducting layers. These results demonstrate the potential of free-standing construction for ultra-thin and lightweight electronics.

### Effect of Strain Anisotropy on Organic TFT Device Performance

**Figure 2:**
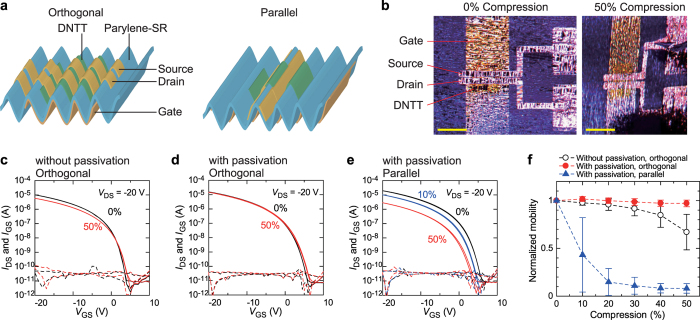
Mechanical robustness of organic TFT devices. (**a**) Illustration of compressive strain induced in the free-standing organic TFT devices. Strain was induced in two directions: (left) orthogonal to the flow of *I*_DS_ (*ε*_⊥_) and (right) parallel to the flow of *I*_DS_ (*ε*_‖_). (**b**) Photos of transistor without compression (left) and under 50% orthogonal compression *ε*_⊥_. Scale bars, 1 mm. (**c–e**) Transfer characteristics (solid lines) and gate leakage current (dashed lines) of TFT devices operated under no strain (black), 10% strain (blue), and 50% strain (red). The device construction and strain directions are as follows: (**c**) without passivation, *ε*_⊥_ (**d**) with passivation *ε*_⊥_ and (**e**) with passivation, *ε*_‖_. (**f**) Change in mobility as a function of compressive strain. The device construction and strain directions are as follows: without passivation and orthogonal compression *ε*_⊥_ (open black circles), with passivation and orthogonal compression *ε*_⊥_ (solid red circles), and with passivation and parallel compression *ε*_‖_ (solid blue triangles). The error bars in the plots indicate the standard deviation.

In order to evaluate how the passivation layer and strain direction affect the mechanical durability of free-standing TFT devices, the stability of the devices under mechanical deformation was evaluated by applying compressive strain. The devices were peeled from their supporting glass plates and laminated onto a pre-stretched elastomer (3 M, VHB Y-4905 J). When the pre-strained elastomer was relaxed, the adhesion between the elastomer and the organic TFT device’s thin film layers caused the strain in the elastomer to transfer to these layers, forming a network of out-of- plane wrinkles in the device. We performed the strain experiment in two directions, orthogonal (*ε*_⊥_) and parallel (*ε*_‖_) to the source-drain current flow (Fig. 2a), whereby the maximum compression was 50% and the device area was reduced by almost 50% of its initial value (Fig. 2b). Almost of all of the devices with 250 nm dielectric layers remained functional even after 50% compression was applied; however, the device yield decreased when the dielectric layer thickness was less than 250 nm (see Table S1 and Fig. S3). Accordingly, devices with total thicknesses of 650 nm were used to assess the mechanical stability of the organic TFT devices, whereby we monitored changes in electrical performance under compressive strain. Figure 2c,d and e show typical transfer characteristics of organic TFT devices of different constructions and strain directions, without passivation and orthogonal strain *ε*_⊥_ (Fig. 2c), with passivation and orthogonal strain *ε*_⊥_ (Fig. 2d), and with passivation and parallel strain *ε*_‖_ (Fig. 2e). Interestingly, there were clear differences in electrical performance between these three experimental conditions. When 50% orthogonal strain *ε*_⊥_ was applied to the devices without passivation layers, the on-current decreased slightly from its initial value (Fig. 2c). On the other hand, the on-current for the devices with passivation layers did not change even when the same amount of strain was applied to the device (Fig. 2d). When parallel strain *ε*_‖_ was applied, a significant decrease was observed even for the devices covered with a passivation layer (Fig. 2e). The on-current level decreased by almost half when the 10% parallel strain *ε*_‖_ was applied and decreased by nearly one order of magnitude when the 50% parallel strain *ε*_‖_ was applied. These decreases were considered irreversible, as the on-current did not return to its initial value after the compressive strain was removed (see Fig. S4). However, the leakage current did not change under these three conditions, which indicates that the dielectric layers did not deteriorate as a result of the compressive strain. Figure 2f shows the change in mobility as a function of compression, whereby the mobility levels were normalized with respect to their initial values (under 0% strain). Note that none of the devices with passivation layers were affected by the orthogonal strain *ε*_⊥_; thereby, the change in average mobility from initial value (Δ*μ*/*μ*_0_) was less than 3% and the standard deviation of the mobility (σ) was quite small (0.03) even when 50% orthogonal strain *ε*_⊥_ was applied. When the devices without the passivation layer were compressed orthogonal to the direction of *I*_DS_, both Δ*μ*/*μ*_0_ and σ increased with compressive strain. When the devices were compressed parallel to the direction of *I*_DS_, even small amounts of strain dramatically affected Δ*μ*/*μ*_0_ and σ, including those of the devices that had a passivation layer. Some devices degraded under 10% parallel strain *ε*_‖_ (Fig. 2f), whereas others remained stable (Fig. S5), which led to a large variation σ (0.35). When the parallel strain *ε*_‖_ was increased to more than 10%, the percentage of degraded devices increased and led to larger Δ*μ*/*μ*_0_ and smaller σ values. Almost of all of the devices deteriorated in performance when 50% parallel strain *ε*_‖_ was applied to them; Δ*μ*/*μ*_0_ was 92%, and σ was 0.04. Unlike the changes in carrier mobility, we were unable to observe specific differences or changes in threshold voltage between the three experimental conditions (see Fig. S6), which indicated that the compressive strain affected the carrier conduction paths but not the carrier accumulation in the semiconducting layer. Additionally, orthogonal compressive stress cycles were applied to the devices with passivation layers. Here, even after 100 full cycles, the TFT devices remained fully functional, and the changes in mobility and threshold voltage amounted to 6.1% and 3.1%, respectively (Fig. S7). This level of stability is comparable to that of the organic TFT devices fabricated on one-micron-thick substrates^2^. Table S2 summarizes the comparison with literature data of free-standing TFTs. This clearly shows that our devices combine extreme bending stability, relative large field-effect mobility and low operating voltage.

### Surface and Cross-Sectional Observations

**Figure 3:**
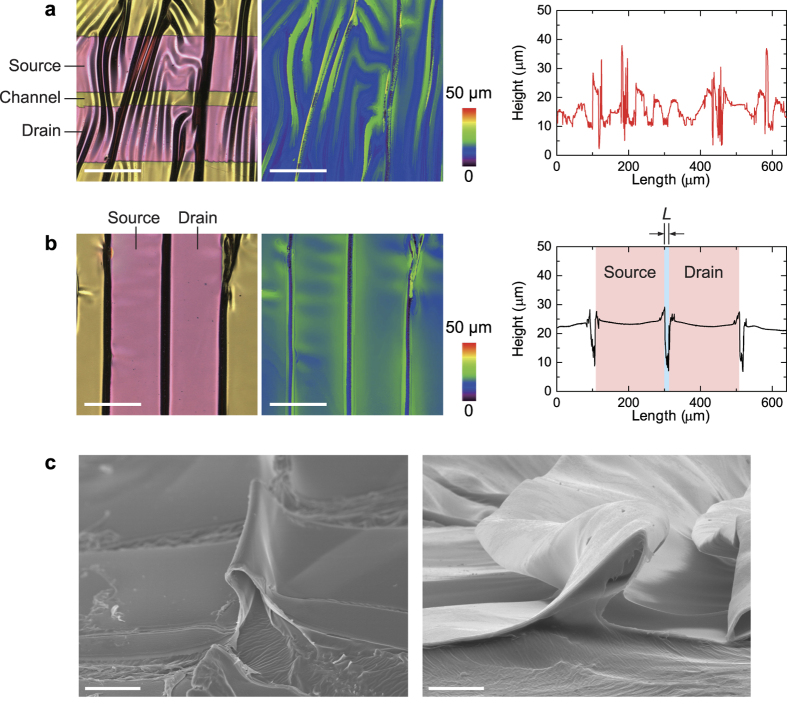
Strain induced surfaces and cross-sections. (**a–d**) Top-view photos (left), height profile (middle), and sectional profile of TFT device near the channel layer under 20% orthogonal compression *ε*_⊥_ (**a**) and 20% parallel compression *ε*_‖_. Scale bars, 200 μm. The surface observations clearly revealed that *ε*_⊥_ induced wrinkles located randomly on the surface, whereas *ε*_‖_ induced wrinkles localized in the channel region (shown as the blue shaded area in the section profiles). (**c**) Cross-sectional SEM image of a device undergoing 20% *ε*_⊥_ (left) and 50% *ε*_⊥_(right). Scale bars, 10 μm.

Several previous reports on TFT devices fabricated on flexible substrates showed similar strain dependences of the carrier mobility on orthogonal strain *ε*_⊥_ and parallel strain *ε*_‖_^2^,^4^, which stands in contrast with the anisotropic changes in mobility that we observed for our free-standing organic TFTs. We attempted to understand what caused such anisotropies by evaluating the surface topology and cross-sectional structures of the devices under compression and by precisely calculating the strain. Figure 3a,b show the surface topologies and section profiles of the devices near the channel region when 20% orthogonal strain *ε*_⊥_ and 20% parallel strain *ε*_‖_ were applied. In particular, when 20% orthogonal strain *ε*_⊥_ was applied, kinks and wrinkles appeared almost randomly across the surface, and they were in the direction parallel to the source-drain current path for spacings of tens of microns (Fig. 3a). On the other hand, localized wrinkles were created when 20% parallel strain *ε*_‖_ was applied. A laser microscopic image (Fig. 3b) clearly shows that virtually no wrinkles appeared on the source-drain electrodes under a relatively small parallel strain *ε*_‖_. Wrinkles orthogonal to the direction of the source-drain current flow appeared at both ends of the source-drain electrodes, resulting in a channel region that was tightly folded, and the spacing between the source and drain decreased from 50 μm to 13 μm. When the devices were compressed tightly, deeper wrinkles randomly formed on the surfaces of the devices (Figs S8 and S9). Cross-sectional images obtained from a scanning electron microscope (SEM) clearly show that even at relatively low levels of compressive strain, the devices were tightly bent, with a very small bending radii of less than 2 μm, and further compression led to nearly folded structures (Fig. 3c).

### Strain Model for Four-Layer Devices

**Figure 4:**
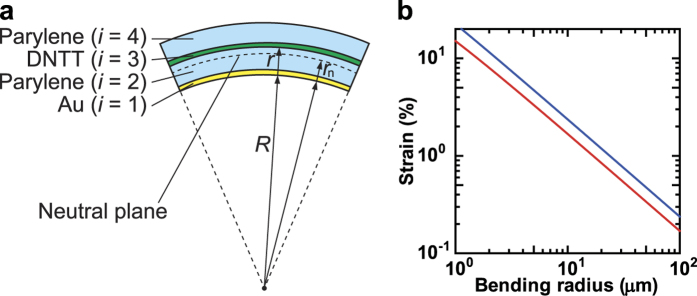
Strain model for a four-layer stacked device. (**a**) Illustration of free-standing organic TFT device under pure bending. The represented layers are a gold gate layer (layer 1: bottom layer), parylene dielectric layer (layer 2: the second from the bottom), DNTT semiconducting layer (layer 3: the third from the bottom), and parylene passivation layer (layer 4: top layer). (**b**) Estimated strain as a function of bending radius using Eq. (1). The blue line represents the strain for the device without passivation (three-layer), and the red line represents the strain for the device with passivation. The resulting equations can be written as follows: *ε* (*h*_1_ + *h*_2_) = (2.38×10^−7^)/(*R* + 6.18×10^−8^) (without passivation) and *ε* (*h*_1_ + *h*_2_) = (1.70×10^−7^)/(*R* + 1.30×10^−7^) (with passivation).

We estimated the bending strain of the organic TFT devices with the 250-nm-thick dielectric layer. Table 1 summarizes the thickness and Young’s modulus of each layer, i.e., Parylene-SR, DNTT and Au, of the devices. We considered the strain of four-layer films, wherein the source/drain electrodes were omitted from the model because the strain applied to the channel layer can significantly affect transistor performance. Figure 4a illustrates the strain model bent into a cylindrical shape of radius *R*, defined at the bottom surface of the device. *h*_*i*_ and *Y*_*i*_ (*i* = 1, 2, 3, 4) are the thicknesses and Young’s moduli for the gate electrode (Au), dielectric layer (Parylene-SR), organic semiconductor layer (DNTT), and passivation layer (Parylene-SR). Assuming that the total thickness of the device, *h* = *h*_1_ + *h*_2_ + *h*_3_ + *h*_4_, is much smaller than the bending radius *R*, the bending strain at an arbitrary position *r* from the bottom surface of the device yields


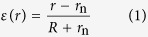


where *r*_n_ denotes the position of the neutral surface^32^. In pure bending, the circumferential stress through the thickness of the device is in mechanical equilibrium, such that 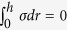. By substituting Hooke’s law *σ* = *Y*_*i*_*ε* into the equilibrium equation and solving it for the neutral position *r*_n_, we obtain:


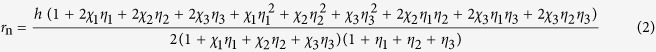


where *η*_1_ = *h*_2_/*h*_1_, *η*_2_ = *h*_3_/*h*_1_, *η*_3_ = *h*_4_/*h*_1_, *χ*_1_ = *Y*_2_/*Y*_1_, *χ*_2_ = *Y*_3_/*Y*_1_, *χ*_3_ = *Y*_4_/*Y*_1_ and we have assumed that the Poisson’s ratios of the layers are identical. The strain at the interface between the dielectric layer (*i* = 2) and the semiconductor layer (*i* = 3), *r* = *h*_1_ + *h*_2_, has a dominant effect on transistor performance:


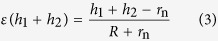


By setting *h*_4_ = Y_4_ = 0 in Eq. (2), the strain for the devices without passivation can be calculated using Eq. (3). We estimated that the neutral surfaces of the devices with or without passivation layers are at 130 nm and 62 nm, respectively. If the total thickness of layers stacked on a substrate is much thinner than the substrate thickness, the thickness *h*_2_ + *h*_3_ + *h*_4_ will be negligible and Eq. (2) reduces to a simple expression^33^, which is widely used to estimate the bending strain for such a device 1,2,34,35,36,37. Nevertheless, our free-standing organic TFT devices cannot ignore the thickness *h*_2_ + *h*_3_ + *h*_4_ because it is comparable to the substrate thickness *h*_1_; *η*_*i*_ (*i* = 1, 2, 3) cannot be approximately set to zero. Therefore, we used Eqs (1) [and (3)] with (2) instead of the approximate formula^33^ to estimate the bending strain.

Figure 4b shows the strain at *r* = *h*_1_ + *h*_2_ calculated using Eq. (3) as a function of bending radius. Though the strain of a four-layer device (with passivation) is smaller than that of a three-layer device (without passivation), the strain could not be eliminated by the passivation layer, as described in previous reports^1^,^9^. The calculated value of *r*_n_ indicates that the neutral position is not located at the interface between the gate dielectric layer and semiconducting layer (*r* = 300 nm), but is within the dielectric layer. This is attributed to the moderately large Young’s modulus of the Au gate layer, which is about five times and 20 times larger than that of the DNTT and parylene-SR layers, respectively, and would prevent the passivation layer from cancelling out the strain. The previous research used such “hard” materials for the gate electrodes, but in those cases, the electrodes were sufficiently thinner than the substrate. The free-standing construction and moderately large Young’s modulus of the bottom layer causes a bias in the neutral strain position. The hardness of the electrodes also causes localized wrinkles when the devices are compressed parallel to the current flow of *I*_DS_ (Fig. 3b). The strain can be easily localized in wrinkles at the edge of the gate electrode and the channel region, which is tightly compressed by relatively small levels of strain. The previous studies reported that organic TFT devices with semiconducting layers made using evaporated small-molecule materials were irreversibly degraded when strain levels of more than 2% were applied to them^34^,^38^. According to Eq. (3) and Fig. 4b, the estimated bending radius corresponding to 2% strain was 12 μm for a three-layer device (without passivation) and 8 μm for a four-layer device (with passivation). A cross-sectional SEM image (Fig. 3c) clearly shows that even small levels of compression formed locally strained areas with bending radii of less than 10 μm and caused irreversible electrical degradation in these areas. When orthogonal strain was applied to the devices, the degraded lengths were considerably smaller than the channel width (1000 μm in this study), such that the change in mobility under compressive strain was also small and could be suppressed by the passivation layer. On the other hand, when parallel strain was applied to the devices, irreversible levels of strain were applied to almost all of the channel region area, which caused a large drop in mobility. The large variations seen with 10% parallel strain *ε*_‖_ imply that some of the devices were not bent to the irreversible bending radii.

### Mechanical Robustness of the Integrated Circuits

**Figure 5:**
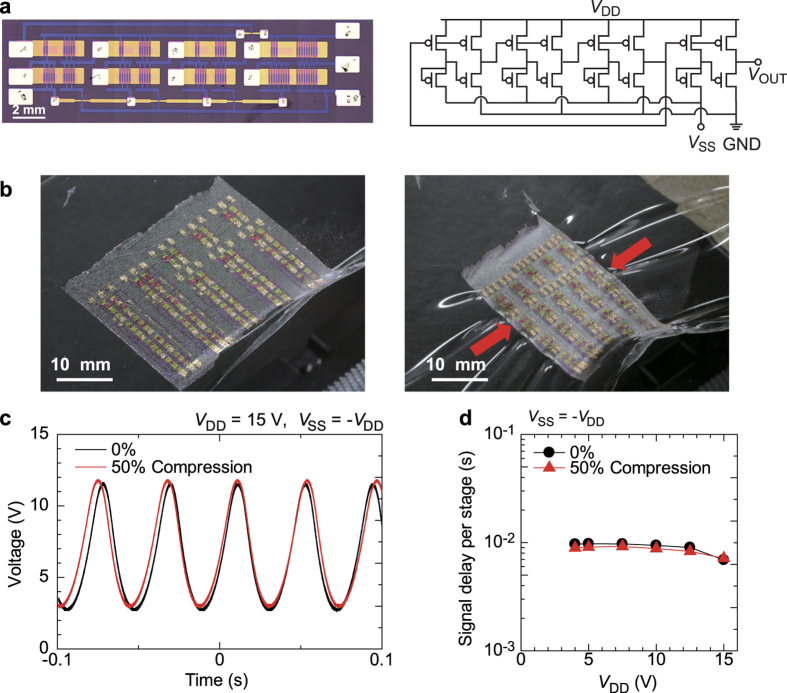
Dynamic response of free-standing organic integrated circuits. (**a**) Photograph (left) and circuit diagram (right) of fabricated three-stage pseudo-CMOS ring oscillator. Scale bar, 2 mm. (**b**) Photos of a three-stage pseudo-CMOS ring oscillator without compression (left) and under 50% compression (right). Scale bars, 10 mm. (**c**) Output signals of the ring oscillator operated with a supply voltage (*V*_DD_) of 15 V and tuning voltage (*V*_SS_) of −15 V under no compression (black) and under 50% orthogonal compression *ε*_⊥_ (red). (**d**) Signal delay per stage obtained from the output signals of the ring oscillator as a function of *V*_DD_. The black solid circles represent the data under no compression, and the red triangles represent the data under 50% orthogonal compression *ε*_⊥_. The operating speeds remained stable and low-voltage operation at 4 V was possible even when a large compressive strain was put on the ring oscillator circuit.

In order to demonstrate the feasibility of the free-standing organic electronic devices, we fabricated three-stage ring oscillators and monitored their electrical performance under compression. The ring oscillators used pseudo-CMOS logic^39^ with a single buffer inverter, resulting in a total of 16 TFT devices (Fig. 5a). To avoid irreversible degradation of the circuits, orthogonal strain *ε*_⊥_ was applied to all TFT devices in the oscillator (Fig. 5b). Figure 5c shows the output signals for an oscillator operated with a supply voltage (*V*_DD_) of 15 V and tuning voltage (*V*_SS_) of −15 V as measured in the flat state and under 50% compression. The amplitude and frequency of the output signals did not change even when the circuits were tightly compressed, likewise for the TFT devices (Fig. 2d) and inverter circuits (Fig. S10). The calculated signal delays per stage were 6.9 ms for devices under no strain and 7.1 ms under 50% compressive strain. The ring oscillators functioned properly at voltages as low as 4 V (Fig. S11). Figure 5b shows the signal delay per stage for the ring oscillator as a function of operation voltage. The oscillators’ operating speed was not affected by the tight compressive strain applied at any operation voltage. These results clearly demonstrate that the free-standing organic integrated circuits exhibited excellent mechanical robustness.

## Conclusion

The fabrication technology described here removes the substrate layer from organic electronic devices to make them ultra-thin and ultra-flexible with total thicknesses of less than one micron. The anisotropic dependency of the devices on the strain directions indicates that these devices require a rigorous strain model that takes in to account the thickness and Young’s modulus of each layer in order to calculate the mechanical strain applied to them. The rigid gold layer may be an important factor affecting the mechanical stability of the devices. Replacement of rigid metal materials with soft materials having smaller Young’s moduli, such as conducting polymers, would improve the mechanical flexibility of these ultra-thin organic electronic devices. Furthermore, such ultra-thin electronics can be stacked on top of one another, which could change the device integration strategy from that of the conventional lateral layout.

## Methods

### Device Fabrication and Characterization

A schematic illustration of a free-standing organic TFT is shown in Fig. 1a, and processing details are shown in Fig. S1. Glass plates (thickness 0.7 mm) were used as supporting carriers, whereby a solution of fluoropolymer (DuPont^TM^, Teflon^®^ AF 1600) in Fluorinert (3M^TM^ FC-43) was spin-coated onto them to form a release layer with a thickness of 80 nm. Next, gold (Au) was thermally evaporated through a shadow mask onto the release layer to form a 50-nm-thick gate electrode. Parylene-SR (KISCO, diX-SR) films were deposited to form a dielectric layer. The thicknesses of the parylene-SR layers ranged from 100 nm to 250 nm. After forming the dielectric layer, the films were heated at 120 °C for 1 hour in ambient air and then slowly cooled to room temperature. DNTT was deposited in a vacuum through a shadow mask to form a 50-nm-thick patterned semiconductor layer on the gate dielectric; the substrate was kept at room temperature during the deposition. Au was evaporated through a shadow mask to form 50-nm-thick source and drain electrodes. The nominal length and width of the channel were 50 and 1000 μm, respectively, for all the organic TFT devices. Finally, some of the devices were uniformly encapsulated with a parylene-SR passivation layer whose thickness was the same as that of the dielectric layer (ranging from 100 nm to 250 nm).

The electrical characteristics of the fabricated capacitors and TFT devices were measured by using a semiconductor parameter analyzer (Keithley, model 4200-SCS). All electrical measurements on the organic TFT devices were carried out in ambient air. The surfaces of the fabricated devices were observed using a laser microscope (Olympus, model OLS-4000) and scanning electron microscope (JEOL, 7600-FE).

## Additional Information

**How to cite this article**: Fukuda, K. *et al.* Free-Standing Organic Transistors and Circuits with Sub-Micron Thicknesses. *Sci. Rep.* **6**, 27450; doi: 10.1038/srep27450 (2016).

## Supplementary Material

Supplementary Information

## Figures and Tables

**Table 1:** Summary of thicknesses and Young’s moduli for each layer.

Layer *i*	Material	Thickness (nm) *h*_*i*_	Young’s modulus (GPa) *Y*_*i*_
4	Parylene-SR	250	4
3	DNTT	50	2.3
2	Parylene-SR	250	4
1	Au	50	79
