# Supplementary material for: Free-Standing Organic Transistors and Circuits with Sub-Micron Thicknesses
Source: Sci Rep. 2016 Jun 9;6:27450. doi: 10.1038/srep27450 (PMC4899690; doi:10.1038/srep27450)
Supplement: Supplementary Information [file srep27450-s1.pdf]

Supplementary Information

Free-standing Organic Transistors and Circuits with Sub-micron thickness

Kenjiro Fukuda[1,2,3,4](*), Tomohito Sekine[1], Rei Shiwaku[1], Takuya Morimoto[5], Daisuke Kumaki[1], and Shizuo Tokito[1](*)

[1]Research Center for Organic Electronics (ROEL), Graduate School of Science and Engineering, Yamagata University

4-3-16 Jonan, Yonezawa, Yamagata, 992-8510, Japan

[2]Japan Science and Technology Agency, PRESTO

4-1-8, Honcho, Kawaguchi, Saitama, 332-0012, Japan

[3]Thin-Film Device Laboratory, RIKEN

2-1, Hirosawa, Wako, Saitama, 351-0198, Japan

[4]Center for Emergent Matter Science, RIKEN

2-1 Hirosawa, Wako-shi, Saitama 351-0198, Japan.

[5]Department of Mechanical, Electrical and Electronic Engineering, Shimane University,

1060 Nishikawatsu, Matsue, Shimane 690-8504, Japan

Address correspondence to: kenjiro.fukuda@riken.jp, tokito@yz.yamagata-u.ac.jp

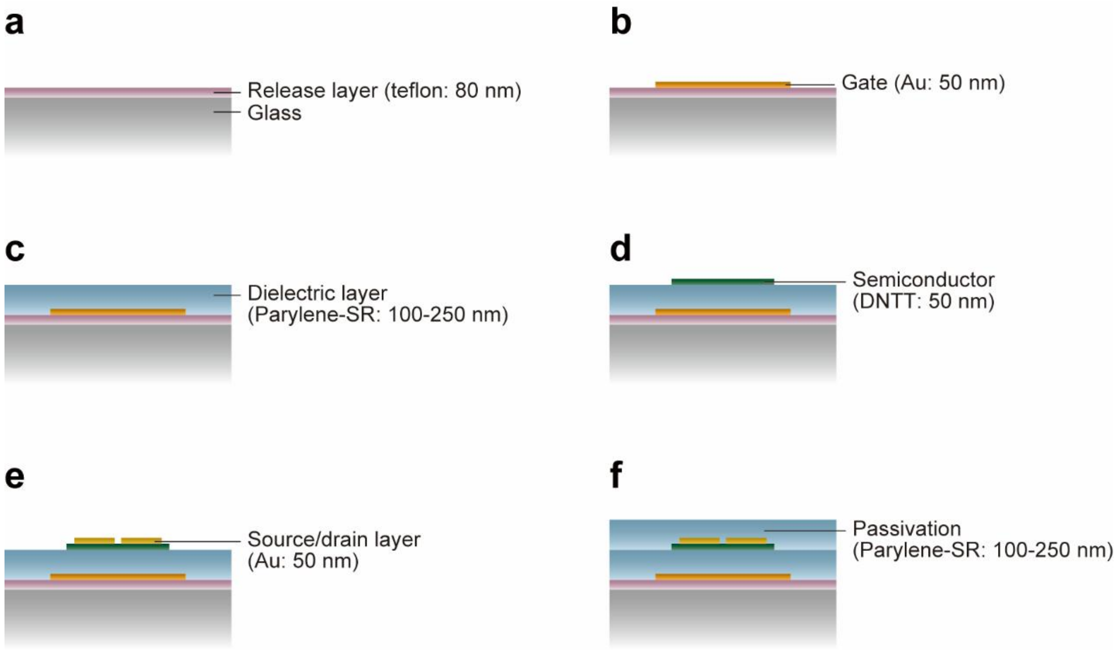

Fig. S1. Fabrication details of substrate-free organic transistors.
(a) Fluoropolymer was spin-coated onto supporting glass plates to form an 80-nm-thick release layer. (b) Gold (Au) was thermally evaporated through a shadow mask to form a 50-nm-thick gate (c) Parylene-SR was deposited to form a dielectric layer. The thickness of the parylene-SR layer was varied from 100 nm to 250 nm. (d) DNTT was deposited in vacuum through a shadow mask to form a 50-nm-thick patterned semiconductor layer on the gate dielectric. (e) Au was thermally evaporated through a shadow mask to form a 50-nm-thick source and drain electrodes. (f) Some of the devices were uniformly encapsulated with a parylene-SR passivation layer whose thickness was the same as that of the dielectric layers (from 100 nm to 250 nm).

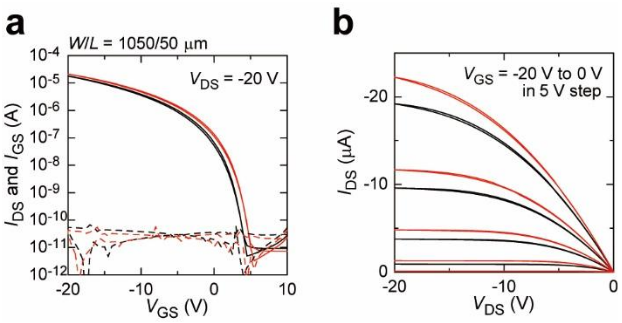

Fig. S2. Transistor characteristics of the device before and after peeling procedure.
The thickness of the dielectric layer was 250 nm, and the device had a 250-nm-thick passivation layer. A photograph of the peeling procedure is shown in Fig. 1b of the main text. (a) Transfer characteristics of an organic TFT device. The plot is of the drain-source current (I_{DS}, solid line) and gate leakage current (I_{GS}, dashed line) as a function of gate-source voltage (V_{GS}) at a drain-source voltage (V_{DS}) of –10 V. Black and red lines represent the characteristics before and after peeling, respectively. (b) Corresponding output characteristics. The plot is of I_{DS} as a function of V_{DS} for V_{GS}

from 0 V to –10 V in 2 V steps.

Table S1. Device yield under 50% compression.

Thickness of dielectric layer [a] (nm)	Yield under 50% compression[b] (%)
250	>90
150	60
100	0

[a]All devices had passivation layers of the same thickness as their dielectric layers.
[b]Strain direction: orthogonal to source-drain current

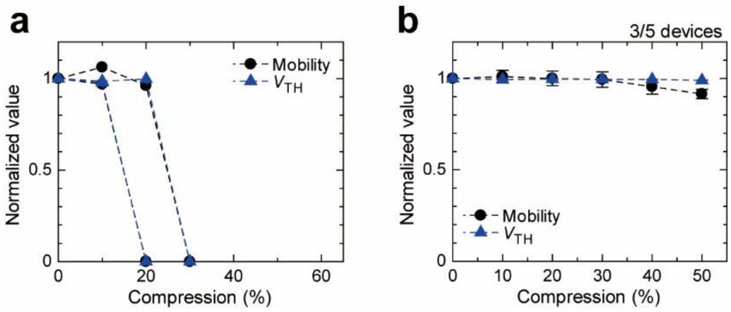

Fig. S3. Performance change under the application of strain for the thinner devices.
Change in mobility as a function of compressive strain for the devices with 100-nm-thick (a) and 150-nm-thick (b) gate dielectric layers. The devices had a passivation layer, and the strain direction is $\varepsilon_{\perp}$. Black circles represent mobility, and blue triangles represent threshold voltage. Mobility and V_{TH} were normalized by their initial (0% strain) values. (a) Results from two devices are plotted. The devices were shorted when strain was applied to them. (b) Three-fifths of the devices were fully functional even when 50% strain was applied to them. The plots show the average values of the functional devices. Error bars indicate s.d. The two-fifths of the devices were shorted when a strain of less than 50% was applied to them. The changes in average mobility ($\Delta\mu/\mu_0$) and V_{TH} were less than 9% and 1%. These results imply that the decrease in device yield under compression was caused by the gate dielectric layers not being sufficiently thick (250 nm). The yields would increase if the process of forming the dielectric layers could be improved to make better parylene-SR layers or more appropriate dielectric materials were used.

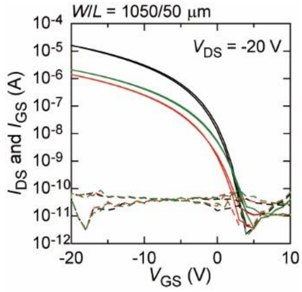

Fig. S4. Irreversible deterioration of the devices under parallel strain.
Transfer characteristics (solid lines) and gate leakage current (dashed lines) of TFT devices operated before strain (black), under 50% $\varepsilon_{\parallel}$ strain (red), and after 50% strain (green). The mobility decreased dramatically from 0.52 to 0.05 cm^2 V^{-1} s^{-1} when strain was applied to the device, and it slightly recovered to 0.06 cm^2 V^{-1} s^{-1} when the strain was lifted from the device.

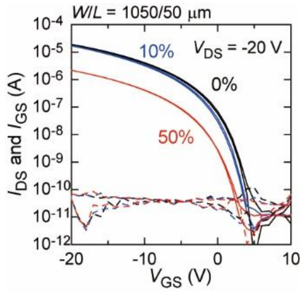

Fig. S5. Large dispersion of the device performance under 10% parallel strain.
Transfer characteristics (solid lines) and gate leakage current (dashed lines) of TFT devices operated under no strain (black), 10% $\varepsilon_{\parallel}$ strain (blue), and 50% $\varepsilon_{\parallel}$ strain (red). Although the fabrication process was completely the same as that of the device shown in Fig. 2e in the main text, there is a big difference in strain sensitivity between these two devices. The mobility only slightly decreased from 0.54 to 0.52 cm^2 V^{-1} s^{-1} (i.e., 4%) when 10% $\varepsilon_{\parallel}$ strain was applied to the device, whereas it dramatically decreased to 0.07 cm^2 V^{-1} s^{-1} when 50% $\varepsilon_{\parallel}$ strain was applied.

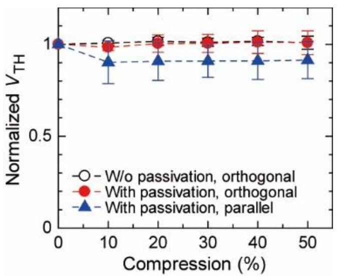

Fig. S6. Changes in threshold voltage.
Changes in threshold voltages are plotted as a function of compressive strain. The device constructions and strain directions are as follows: without passivation and $\varepsilon_{\perp}$ (open black circles), with passivation and $\varepsilon_{\perp}$ (solid red circles), and with passivation and $\varepsilon_{\parallel}$ (solid blue triangles). Error bars indicate s.d.

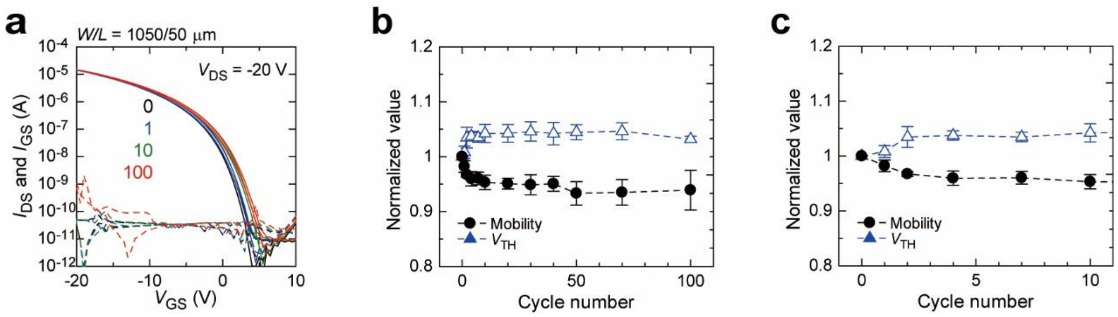

Fig. S7. Strain cycle test.
(g) Mechanical durability during repeated application of 50% $\varepsilon_{\perp}$ to the TFT devices with passivation. The transfer characteristics (solid lines) and gate leakage current (dashed lines) are plotted as a function of V_{GS} at a V_{DS} of –10 V. Black lines represent before the cycle test. The other curves were obtained after 1, 10, and 100 cycles. (h) Changes in electrical performance as a function of strain cycles. Mobility (solid black circles) and threshold voltage (blue open triangles) are plotted as a function of stress cycles. Error bars indicate s.d. When the strains were applied up to ten times to the devices, the threshold voltage shifted in the negative direction and mobility decreased slightly (about 5%). Applying more than ten cycles did not change the performance of the devices.

Table S2: Comparison with literature data of free-standing TFTs.

Dielectric material	Dielectric thickness (nm)	Total thickness (nm)	V_{GS} (V)	Mobility ($cm^2 V^{-1} s^{-1}$)	Critical strain	ref.
PET (Mylar™)	900	N/A	–100	10^{-4}	N/A	[13]
PET (Mylar™)	900	N/A	–100	N/A	N/A	[14]
PET (Mylar™)	900	N/A	–90	5×10^{-4}	N/A	[15]
PET (Mylar™)	1900	N/A	–100	2×10^{-2}	N/A	[16]
PET (Mylar™)	1600	N/A	–100	10^{-3}–10^{-2}	N/A	[17]
PET (Mylar™)	1600	1850	–100	10^{-3}–10^{-2}	N/A	[18]
PET (Mylar™)	1600	N/A	100	3.5×10^{-5}	N/A	[19]
PET (Mylar™)	1600	N/A	–100	3.5×10^{-2}	N/A	[20]
Parylene-C	400	500-550	–20	4.0×10^{-2}	R < 2 mm	[21]
PET (Mylar™)	2500	N/A	–50	0.4	R < 0.1 mm	[22]
PET (Mylar™)	1500	N/A	–100	5.0×10^{-2}	N/A	[23]
Polyacrylonitriile /polystyrene	320	N/A	–15	0.53	R = 5 μm	[24]
Poly(methylmethacrylate)	500	600	–60	0.3	N/A	[25]
Polystyrene	600	770	–80	0.11	R < 1 mm	[26]
polylactide	8000	>10000	–50	1.27×10^{-6}	N/A	[27]
polylactide	5000	5300	–80	0.58	R < 800 μm	[28]
Parylene-SR	100	350[a)]	–10	0.37	20% compression[c)]	This study
Parylene-SR	250	400[b)]	–20	0.45	>50% compression (R < 2 μm)[c)]	This study
Parylene-SR	250	650[a)]	–20	0.36	>50% compression (R < 2 μm)[c)]	This study
Parylene-SR	250	650[a)]	–20	0.53	10% compression[d)]	This study

[a)]With passivation layer. [b)]Without passivation layer. [c)]Orthogonal strain. [d)]Parallel strain.

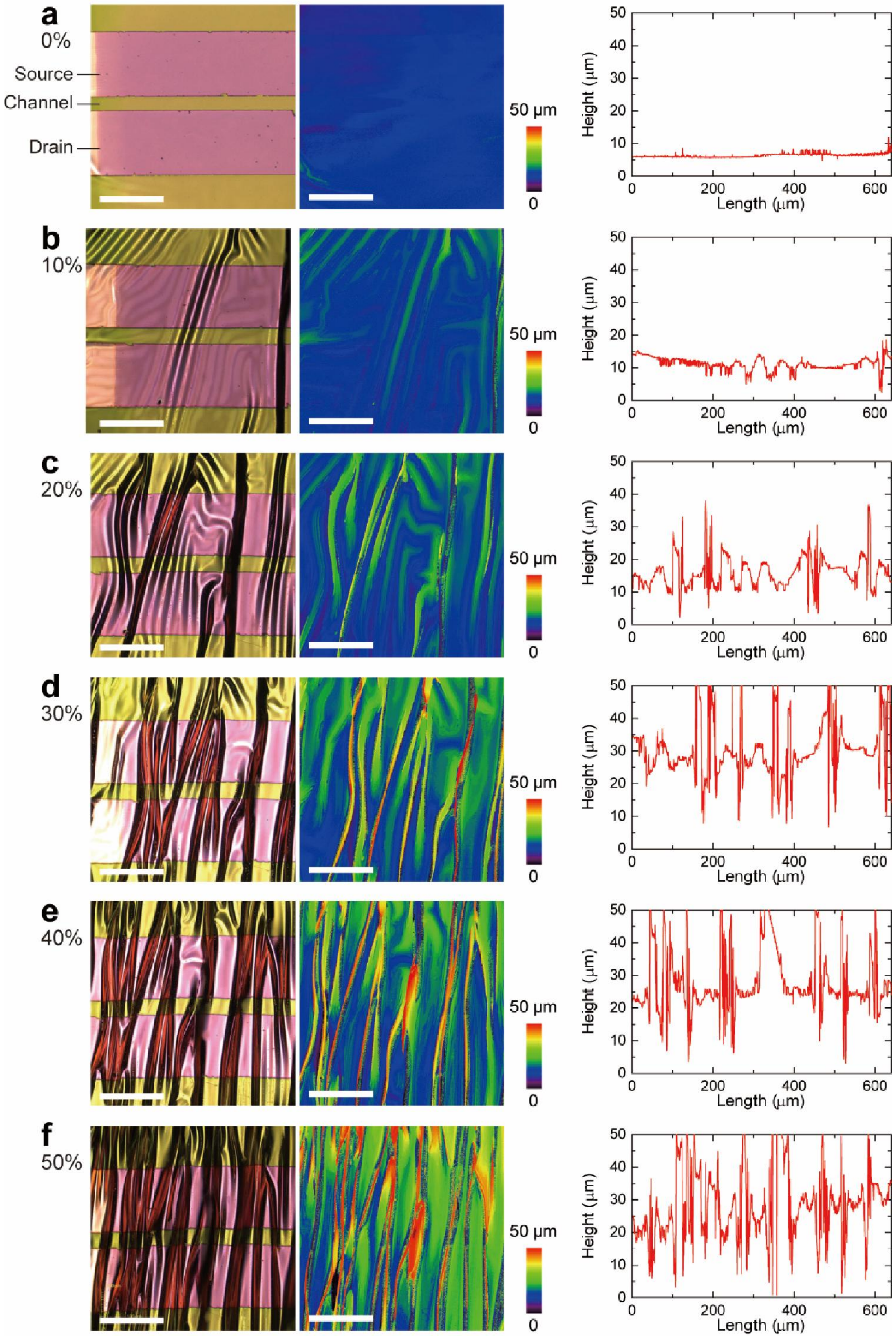

Fig. S8. Orthogonal compressive strain.

Top-view photos (left), height images (middle), and section profile of a TFT device near the channel layer without strain (a), under 10% (b), 20% (c), 30% (d), 40% (e), and 50% (f) $\varepsilon_{\perp}$. Scale bars, 200 μm. The laser microscopic images show that wrinkles randomly appeared on the surfaces. The number and depth of the wrinkles became larger as the strain increased.

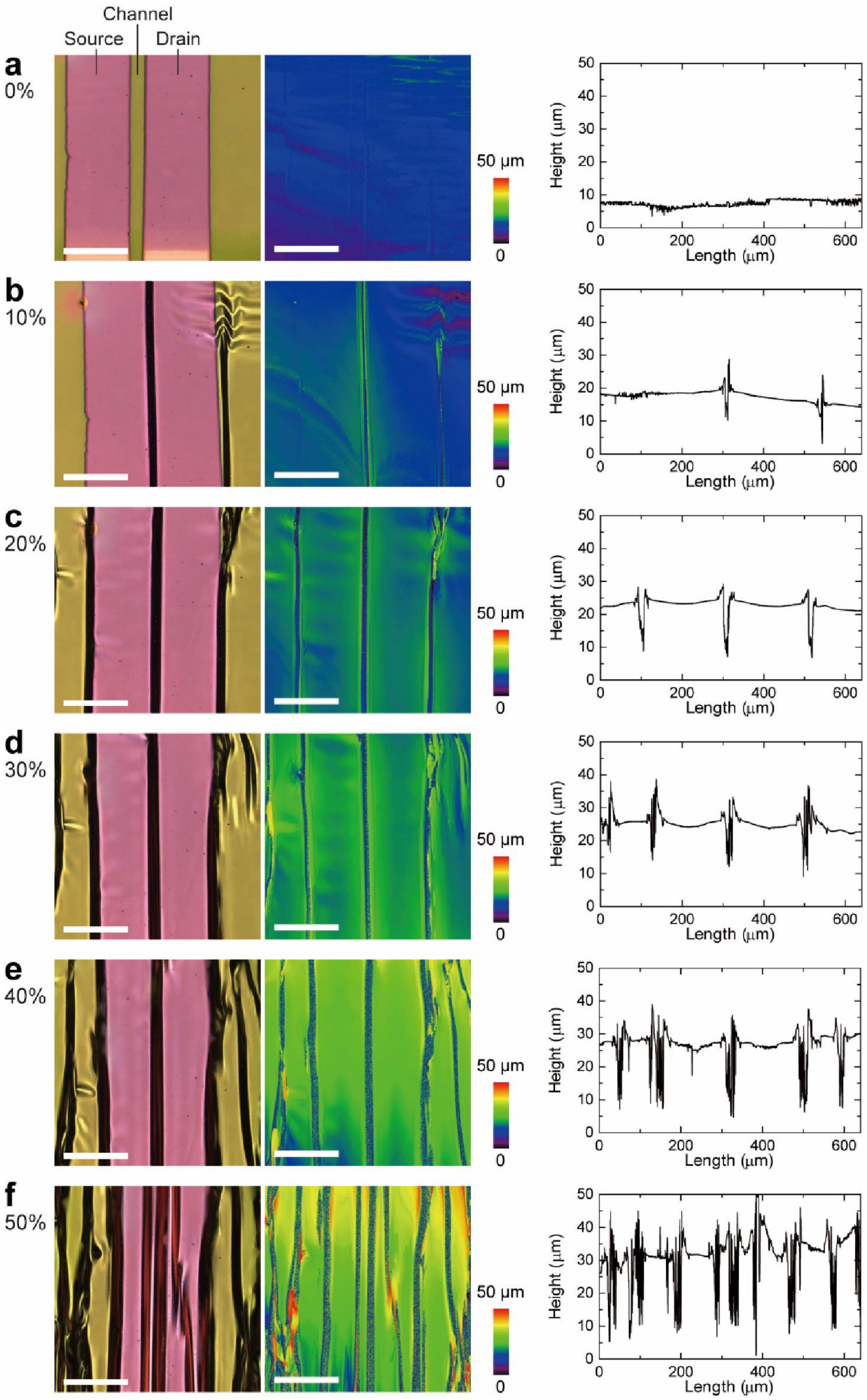

Fig. S9. Parallel compressive strain.

Top-view photos (left), height images (middle), and section profile of a TFT device near the channel layer without strain (a), under 10% (b), 20% (c), 30% (d), 40% (e), and 50% (f) $\varepsilon_{\parallel}$. Scale bars, 200 µm. The laser microscopic images show that a small strain (less than 50%) did not cause wrinkles on the surfaces of the source-drain electrodes. Therefore, the channel region was tightly folded even under a small compression. When 50% strain was applied, wrinkles appeared on the surfaces of the source-drain electrodes.

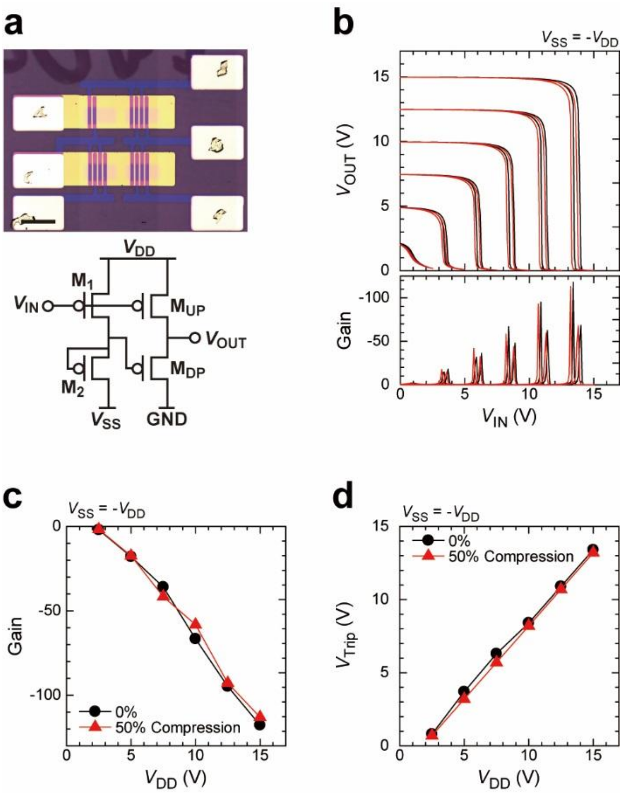

S10. Mechanical robustness of inverter circuits.

(a) Photograph (top) and circuit diagram (bottom) of fabricated pseudo-CMOS inverter circuit. Scale bar, 10 mm. (b) Input-output signals of the inverter. The output voltage and small-signal gain are plotted as a function of input voltage (V_{IN}). The supply voltages (V_{DD}) were set from 2.5 V to 15 V in 2.5 V steps and the tuning voltage V_{SS} equaled $-V_{DD}$. Black and red lines represent data under no compression and under 50% $\varepsilon_{\perp}$, respectively. (c) Small-signal gain and (d) Trip voltage (V_{Trip}) as a function of V_{DD}. Black and red lines represent data under no compression and under 50% $\varepsilon_{\perp}$, respectively. The static characteristics clearly show that the inverter characteristics did not change even when the devices were tightly compressed.

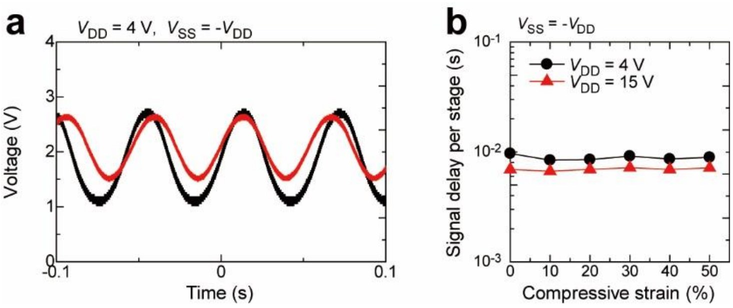

S11. Low-voltage operation of ring-oscillator.

(a) Output signals of the ring oscillator operated with a supply voltage (V_{DD}) of 4 V and tuning voltage (V_{SS}) of –4 V under no compression (black) and under 50% $\varepsilon_{\perp}$ (red). (b) Signal delay per stage obtained from the output signals of the ring oscillator as a function of compressive strain. Black circles represent data at V_{DD} = 4 V, and red triangles represent data V_{DD} = 15 V. The signal delays were almost independent of the compressive strain for small and large operation voltages.
